# Phylogenetic Reconstruction, Morphological Diversification and Generic Delimitation of *Disepalum *(Annonaceae)

**DOI:** 10.1371/journal.pone.0143481

**Published:** 2015-12-02

**Authors:** Pui-Sze Li, Daniel C. Thomas, Richard M. K. Saunders

**Affiliations:** School of Biological Sciences, The University of Hong Kong, Pokfulam Road, Hong Kong, P. R. China; Saint Mary's University, CANADA

## Abstract

Taxonomic delimitation of *Disepalum* (Annonaceae) is contentious, with some researchers favoring a narrow circumscription following segregation of the genus *Enicosanthellum*. We reconstruct the phylogeny of *Disepalum* and related taxa based on four chloroplast and two nuclear DNA regions as a framework for clarifying taxonomic delimitation and assessing evolutionary transitions in key morphological characters. Maximum parsimony, maximum likelihood and Bayesian methods resulted in a consistent, well-resolved and strongly supported topology. *Disepalum s*.*l*. is monophyletic and strongly supported, with *Disepalum s*.*str*. and *Enicosanthellum* retrieved as sister groups. Although this topology is consistent with both taxonomic delimitations, the distribution of morphological synapomorphies provides greater support for the inclusion of *Enicosanthellum* within *Disepalum s*.*l*. We propose a novel infrageneric classification with two subgenera. Subgen. *Disepalum* (= *Disepalum s*.*str*.) is supported by numerous synapomorphies, including the reduction of the calyx to two sepals and connation of petals. Subgen. *Enicosanthellum* lacks obvious morphological synapomorphies, but possesses several diagnostic characters (symplesiomorphies), including a trimerous calyx and free petals in two whorls. We evaluate changes in petal morphology in relation to hypotheses of the genetic control of floral development and suggest that the compression of two petal whorls into one and the associated fusion of contiguous petals may be associated with the loss of the pollination chamber, which in turn may be associated with a shift in primary pollinator. We also suggest that the formation of pollen octads may be selectively advantageous when pollinator visits are infrequent, although this would only be applicable if multiple ovules could be fertilized by each octad; since the flowers are apocarpous, this would require an extragynoecial compitum to enable intercarpellary growth of pollen tubes. We furthermore infer that the monocarp fruit stalks are likely to have evolved independently from those in other Annonaceae genera and may facilitate effective dispersal by providing a color contrast within the fruit.

## Introduction

The Southeast Asian genus *Disepalum* Hook. f. (Annonaceae subfam. Annonoideae tribe Annoneae [1]) consists of nine species of shrubs and small trees [2]. Despite its small number of species, the genus exhibits a striking diversity in floral morphology and is an ideal candidate for studying the evolutionary diversification of floral structures and their functional significance.

**Fig 1:**
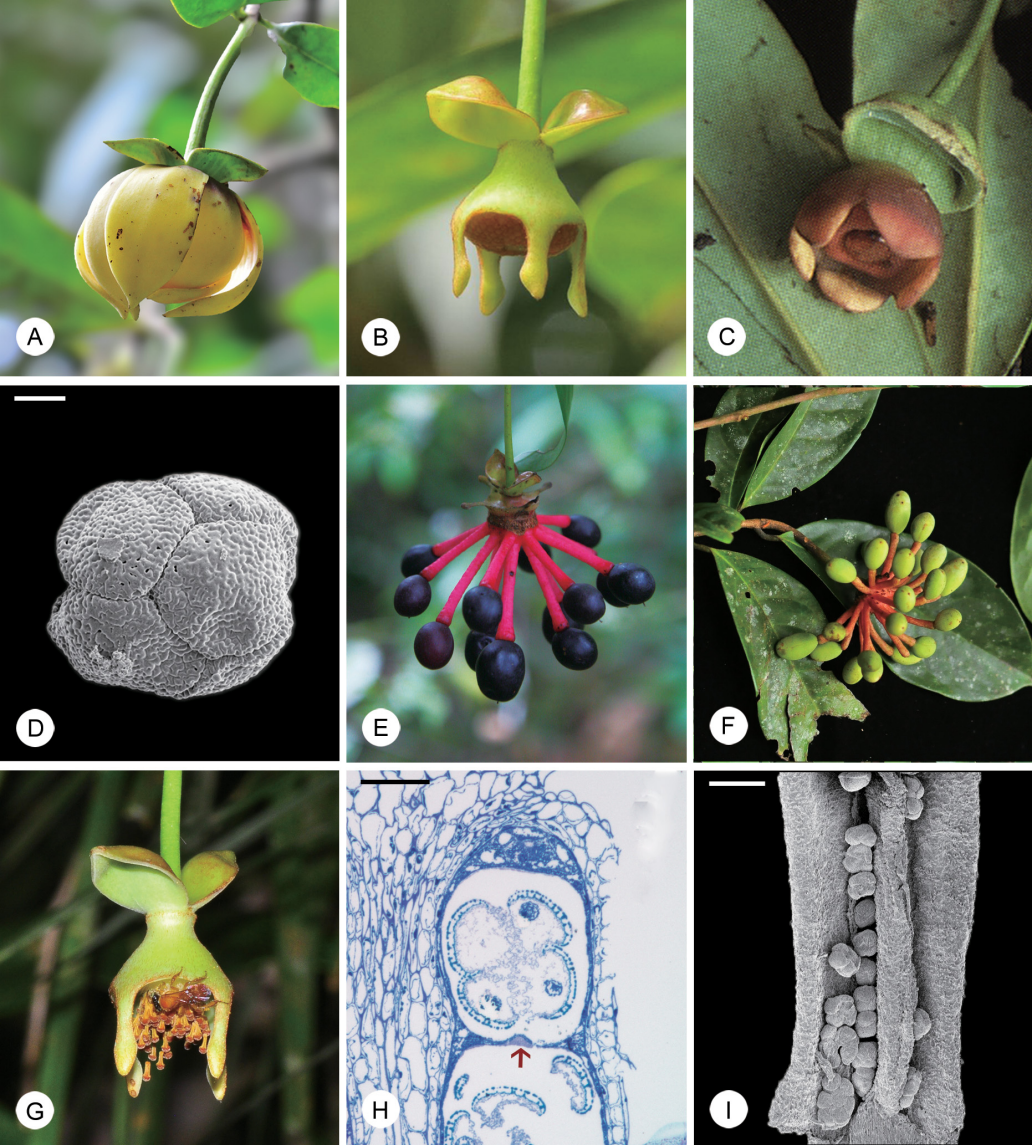
Flowers, fruits and pollen of selected *Disepalum* species. (A) Flower of *Disepalum pulchrum*. (B) Flower of *Disepalum anomalum*. (C) Flower of *Disepalum platypetalum*. (D) Pollen octad of *Disepalum petelotii* (*Pételot 5471*, A; scanning electron micrograph). (E) Mature fruit of *Disepalum anomalum*. (F) Immature fruit of *Disepalum pulchrum*. (G) Staminate flower of *Disepalum anomalum*, visited by meliponine bee. (H) Immature theca of *Disepalum anomalum* (longitudinal section), showing septum (arrowed) between thecal chambers (*P*.*S*. *Li LPS1*, HKU). (I) Dehisced anther of *Disepalum petelotii* (*Pételot 5471*, A; scanning electron micrograph). Photographs: (C) Mitsuru Hotta, reproduced from [5], courtesy of Research Center for the South Pacific, Kagoshima University; (E), courtesy of Chin Cheung Tang. Scale bars: D, H = 25 μm; I = 200 μm.

*Disepalum* is divisible into two morphological groups. The first group consists of three species that were previously classified in the genus *Enicosanthellum* Bân [3], with a trimerous perianth consisting of a calyx of three sepals and two whorls of three petals (e.g., *Disepalum pulchrum* (King) J. Sinclair: Fig 1A), a floral type that is typical of the family [4]. The second group, equivalent to *Disepalum s*.*str*., consists of six species with an aberrant floral structure comprising a calyx of two sepals and a single whorl of connate petals (e.g., *D*. *anomalum* Hook. f.: Fig 1B). Among the six species in *Disepalum s*.*str*., *D*. *platypetalum* Merr. (Fig 1C) possesses flowers that are superficially intermediate between the two groups [2]: it has only one whorl of connate petals (cf. *Disepalum s*.*str*.) and yet some flowers have both free and connate petals. The ‘*Enicosanthellum* group’ is distributed in montane forests (600–1900 m altitude) in continental Asia, whereas *Disepalum s*.*str*. is largely confined to tropical lowland forests (generally sea level to 1000 m, although *D*. *platypetalum* extends to 2500 m) in western Malesia [2].

The generic name *Disepalum* was established with the description of *D*. *anomalum*, which has two sepals per flower (Fig 1B) [6], in contrast with most other Annonaceae species that have a trimerous calyx [4]. Five other species were subsequently added to the genus as they share this unusual sepal number and because of their distinctive connate corolla ([2] and references therein).

The three species in the ‘*Enicosanthellum* group’ were originally classified in the genus *Polyalthia* as *P*. *petelotii* Merr. [7], *P*. *plagioneura* Diels [8] and *P*. *pulchra* King [9]. *Polyalthia* in its traditional delimitation is now known to have been highly polyphyletic ([10] and references therein), although the removal of these three species was only a small step towards rendering the genus monophyletic. *Polyalthia pulchra* was transferred to *Disepalum* on the basis of similarities in leaf, stamen, carpel and fruit morphology [11], whereas *P*. *petelotii* and *P*. *plagioneura* were transferred [2] based on the common possession of large pollen octads (Fig 1D), which are diagnostic for *Disepalum* [12]. Taxonomic consensus over these changes was not achieved, however: Bân [3] opted instead to transfer *P*. *petelotii* and *P*. *plagioneura* to a newly described genus, *Enicosanthellum*, to which Maas et al. [13] subsequently added *P*. *pulchra*. Taxonomic opinion regarding the delimitation of *Disepalum* therefore differs: some researchers have adopted a narrow delimitation of *Disepalum* (six species), segregated from the genus *Enicosanthellum* (three species) [3,13]; whereas others have adopted a broader circumscription of *Disepalum* (nine species), inclusive of *Enicosanthellum* [2,11,12].

Johnson [2] published a comprehensive description of all nine species, including a morphology-based phylogenetic analysis that was used to support the broader generic delimitation with explicit reference to morphological synapomorphies. *Disepalum s*.*l*. was shown to be characterized by monocarps borne on elongated ‘carpophores’ (e.g., *D*. *anomalum*: Fig 1E; *D*. *pulchrum*: Fig 1F) that are extensions of the floral receptacle, in contrast with monocarp ‘stipes’ (extensions of the base of the fertilized carpel) that are more common in the Annonaceae, including most other members of tribe Annoneae [14]. Johnson [2] also highlighted pollen octads (e.g., *D*. *petelotii* (Merr.) D.M. Johnson: Fig 1D) as a synapomorphy for *Disepalum s*.*l*. Ancestral character reconstructions [15] reveal that pollen polyads are derived in the Annonaceae, having evolved independently in several disparate lineages. The tribe Annoneae is characterised by polyads, with a transition apparent from tetrads (most genera) to octads (in *Disepalum* [2,12]). In addition to identifying morphological synapomorphies for *Disepalum s*.*l*., Johnson [2] also identified synapomorphies for *Disepalum s*.*str*., viz. reduction of the calyx to only two sepals, connation of petals, and adnation of the petals to the receptacle. Corresponding synapomorphies for *Enicosanthellum* were significantly lacking.

Johnson’s phylogenetic analysis [2] was based on only seven morphological characters, which was insufficient for satisfactorily resolving relationships within the genus. Outgroup selection was also problematic due to an inadequate understanding of higher-level relationships in the Annonaceae prior to the application of molecular phylogenetic methods. Recent molecular phylogenetic studies of the family [1,16,17,18,19, 20] consistently recover *Asimina* as sister to *Disepalum*, with the two genera collectively sister to *Annona*. Species-level phylogenetic relationships within *Disepalum* remain inadequately resolved.

The primary aim of the present study is therefore to reconstruct the phylogeny of *Disepalum* and related taxa based on sequences of selected chloroplast and nuclear DNA regions. The resultant topology is used here to assess the monophyletic status of *Disepalum s*.*l*., the ‘*Enicosanthellum* group’ and *Disepalum s*.*str*., thereby enabling a re-evaluation of the prevailing but contrasting taxonomic opinions regarding generic delimitation.

The presence of intermediate floral forms of *D*. *platypetalum* might imply that it is of recent hybrid origin or part of a lineage derived from an ancient hybridization event. Hybridization refers to crossing between species or genetically diverse populations, resulting in a range of genetic combinations and intermediate morphologies [21]. Evidence for hybridization can be provided by topological incongruence between phylogenies based on chloroplast and nuclear sequence data [22] since the chloroplast and nuclear genomes are maternally and biparentally inherited, respectively; we therefore aim to identify possible incongruence to assess the putative hybrid origin of *D*. *platypetalum*.

Species-level phylogenetic reconstruction of *Disepalum* also enables an assessment of evolutionary transitions in morphological characters of particular taxonomic and functional significance by mapping character state changes onto the phylogeny. The results of the ancestral state reconstructions are used to evaluate hypotheses relating to three functional aspects: (a) whether loss of the floral chamber due to changes in perianth morphology (including petal number, petal whorl arrangement and petal connation) is likely to be functionally associated with a shift in pollination system, or whether the loss is functionally neutral; (b) whether pollen aggregation is associated with an increase in the number of carpels per flower, possibly in response to unfavorable pollination conditions; and (c) whether the evolution of carpophores facilitates exposure of monocarps to frugivores, possibly associated with a shift in seed dispersal mechanism within the tribe Annoneae.

## Materials and Methods

### Taxon sampling

The ingroup for the phylogenetic analyses consisted of eight *Disepalum* species (all species in the genus except *D*. *acuminatissimum* Boerl. & Koord., which is only known from fragmentary type material). Three specimens of *D*. *platypetalum* were sampled to ensure that both of the distinct floral types displayed by this species were included in the study: two of these samples (‘*D*. *platypetalum* I’) possessed the *Disepalum s*.*str*. floral type, and the other (‘*D*. *platypetalum* II’) possessed the intermediate floral type between *Disepalum s*.*str*. and the ‘*Enicosanthellum* group’.

The outgroup consisted of 22 species, including ten species from six other genera in the tribe Annoneae (*Annona*, *Anonidium*, *Asimina*, *Diclinanona*, *Goniothalamus* and *Neostenanthera*), supplemented by representative species from phylogenetically disparate genera in: subfam. Annonoideae tribes Duguetieae (*Duguetia*), Guatterieae (*Guatteria*), Monodoreae (*Monodora*), Uvarieae (*Uvaria*), and Xylopieae (*Xylopia*); subfam. Malmeoideae tribe Miliuseae (*Polyalthia*, *Stenanona* and *Neo-uvaria*); subfam. Ambavioideae (*Ambavia*); and subfam. Anaxagoreoideae (*Anaxagorea*) (subfamilial and tribal classification according to Chatrou et al. [1]). The sample voucher information and GenBank accession numbers are provided in S1 File.

### DNA extraction, amplification and sequencing

Total genomic DNA was extracted from silica-dried leaf material and herbarium specimens using DNeasy Plant Mini Kits (Qiagen) or a modified cetyl trimethyl ammonium bromide (CTAB) method [23,24,25]. DNA extracted by the modified CTAB method was purified using the Wizard PCR Preps DNA Purification System (Promega, Madison, Wisconsin) and eluted with 50 μl pre-heated TE (Tris-EDTA) buffer.

Four chloroplast DNA regions, *matK*, *trnL-F*, *ycf1* and *ndhF*, and two low-copy nuclear DNA regions, *AP3* (MADS domain) and *phyA* (exon 1), were sequenced in this study. The following primers were used: *matK* (matK-424F, matK-788R, matK-13F, and matK-515R [25]); *trnL-F* (trnLF-13F, trnLF-578R, trnLF-413F, and trnLF-774R [25]); *ndhF* (1F, 972R, 2110R, and 972F [26]; 561F, 584R, and 1216F [19]; LBCF, LBCR, and 689R [27]; c2aF, c3bR, c4bR, and c5bF [28,29]; and s8bR [30]); *phyA* (PhyA-F48, PhyA-R350, PhyA-F259, PhyA-R610, PhyA-F563, and PhyA-R954 [X. Guo, unpubl.]); and *ycf1* (Magnoliid3570Fycf1 and Magnoliid5577Rycf1 [31]; and M1426F and M1792R [19]). Novel internal primers (S2 File) were designed as DNA extracted from herbarium materials was likely to be degraded and contain PCR-inhibiting compounds [32,33]. Each 25 μl PCR reaction consisted of 1x PCR buffer, 3 mM MgCl_2_, 0.5 μg/μl bovine serum albumin (BSA) (Promega, Madison, Wisconsin, USA), 0.2 mM dNTP, 0.3 μM of each forward and reverse primer, 1U Taq DNA polymerase (Promega, Madison, Wisconsin, USA), and c. 10 ng DNA template. The names and sources of all primers involved in this study are summarized in S2 File, and details of PCR protocols are presented in S3 File.

PCR products were purified with the QIAquick PCR purification kit (Qiagen Inc., Valencia, California, USA), following the manufacturer’s instructions. DNA was commercially sequenced based on both forward and reverse strands with BigDye Terminator Cycle Sequencing Kit (Applied Biosystems, Foster City, California, USA) using an Applied Biosystems 3730XL DNA Analyzer.

### Alignment and molecular phylogenetic analyses

Sequence data were assembled and edited using Geneious 5.4.3 [34]. Sequences were pre-aligned by the MAFFT [35] plugin in Geneious with automatic algorithm selection and default settings, followed by manual checking and optimization. Ninety-eight ambiguously aligned characters from the *trnL-F* region were excluded from the analyses. The concatenated dataset consisted of a total of 7,358 aligned characters (*matK*: 831 bp; *trnL-F*: 877 bp; *ndhF*: 2,057 bp; *ycf1*: 2,109 bp; *AP3*: 602 bp; *phyA*: 882 bp; Table 1). Descriptive statistics for the concatenated dataset and each partition, including the number and proportion of excluded sites, missing data, variable sites and parsimony-informative sites, are provided in Table 1.

**Table 1:** Descriptive statistics for each of the four cpDNA and two nDNA regions and the concatenated datasets, including the number and proportion of excluded sites, missing data, variable sites and parsimony-informative sites.

DNA region	Alignment length	Excluded sites	Missing data (%)		Variable characters (%)				Parsimony-informative characters (%)			
			Entire dataset	Ingroup	Entire dataset		Ingroup		Entire dataset		Ingroup	
**Chloroplast DNA data**												
*matK*	831	0	6.5	13.3	254	(30.6)	16	(1.9)	146	(17.6)	9	(1.1)
*trnL-F*	877	98	7.5	13.7	255	(29.1)	29	(3.3)	120	(13.7)	12	(1.4)
*ndhF*	2057	0	11.3	7.6	764	(37.1)	61	(3.0)	473	(23.0)	34	(1.7)
*ycf1*	2109	0	46.8	8.4	558	(26.5)	68	(3.2)	503	(23.9)	35	(1.7)
Combined data	5874	98	22.8	9.6	1831	(31.2)	174	(3.0)	1242	(21.1)	90	(1.5)
**Nuclear DNA data**												
*AP3*	602	0	73.3	34.5	160	(26.6)	42	(7.0)	113	(18.8)	14	(2.3)
*phyA*	882	0	82.8	45.1	72	(8.2)	21	(2.4)	11	(1.2)	9	(1.0)
Combined data	1484	0	79.0	40.8	232	(15.6)	63	(4.2)	124	(8.4)	23	(1.5)
**Combined chloroplast and nuclear data**												
	7358	98	34.1	15.9	2063	(28.0)	237	(3.2)	1366	(18.6)	113	(1.5)

Phylogenetic reconstruction was achieved using three methods: maximum parsimony (MP), maximum likelihood (ML) and Bayesian inference (BI). MP analyses of combined sequences from four chloroplast and two nuclear DNA regions were performed using PAUP* 4.0b10 [36]. All characters were treated as unordered, independent, and of equal weight, with gaps as missing data. The most parsimonious trees were obtained by a heuristic search of 1000 replicates of random stepwise sequence addition and tree bisection-reconnection (TBR) branch swapping, with an unlimited number of trees saved. Bootstrap clade support was calculated using 1000 random-addition-sequence replicates with TBR branch swapping, with 10 trees saved per replicate.

ML analyses were performed on both unpartitioned and partitioned datasets, with partitioning schemes including: (a) two partitions distinguishing chloroplast DNA (cpDNA: *matK*, *trnL-F*, *ndhF* and *ycf1*) and nuclear DNA (nDNA: *AP3* and *phyA*) regions; and (b) six partitions based on DNA region identity. ML analyses were conducted using NSF teragrid applications of RAxML 7.3.2 [37] provided by CIPRES Science Gateway [38]. Fifty inferences were run from distinct random-stepwise-addition-sequence MP starting trees under the general time-reversible nucleotide substitution model with among-site rate variation modeled with gamma distribution (GTR+Γ). Subsequently, 1000 non-parametric bootstraps were performed under the partitioned data mode.

The Bayesian reconstructions were performed on both unpartitioned and partitioned datasets, with the same partitioning schemes as described above for the ML analyses. The best-fitting nucleotide substitution models and gamma rate heterogeneity for the unpartitioned dataset and each partition (Table 2) were selected based on the Akaike Information Criterion [39] in MrModelTest 2.3 [40]. The BI analyses were conducted using MrBayes 3.1.2 [41]. Two independent runs, each including four Metropolis-coupled Markov chain Monte Carlo (MCMCMC) analyses, were run for 5 million generations and sampled every 1,000 generations for each partition scheme. Three incrementally heated and one cold Markov chains were used in each run (nchains = 4), with a temperature parameter setting of 0.1. Overall performance of the analyses was assessed in Tracer 1.5 [42] to determine whether the MCMCMC parameter samples were drawn from a stationary, unimodal distribution and whether adequate effective sample sizes (ESS) for each parameter (ESS > 200) were reached. The final average standard deviation of split frequencies was used as the convergence index, in which values < 0.005 indicated good convergence, and convergence of clade posterior probabilities within and between runs were checked using the Cumulative and Compare functions of AWTY [43]. The initial 25% of the sampled trees for each MCMCMC run were discarded as burn-in, and the post-burn-in trees were used to generate a 50% majority-rule consensus tree. Performance of the two partitioning strategies was assessed by Bayes factor comparison implemented in Tracer 1.5 [42], with a 2ln Bayes factor of ≥ 10 interpreted to indicate strong evidence in favor of one strategy over another [44].

**Table 2:** Best-fitting models and parameter values for separate and combined datasets.

DNA region	AIC model selected	AIC	Base frequencies				Substitution model (rate matrix)						Ti/tv	I	G
			A	C	G	T	A-C	A-G	A-T	C-G	C-T	G-T			
**Chloroplast DNA data**															
*matK*	GTR+Γ	6442.2	0.29	0.19	0.17	0.35	1.24	5.19	0.46	0.80	3.89	1.00	–	0.37	–
*trnL-F*	GTR+Γ	6658.2	0.34	0.19	0.18	0.29	0.78	1.70	0.46	0.39	1.97	1.00	–	0.00	0.66
*ndhF*	GTR+Γ+I	21240.4	0.27	0.17	0.18	0.38	1.80	7.34	0.75	1.63	5.52	1.00	–	0.28	0.92
*ycf1*	GTR+Γ	12275.2	0.42	0.15	0.17	0.26	1.39	1.82	0.33	1.36	1.58	1.00	–	0.00	0.60
Combined data	GTR+Γ+I	48201.1	0.33	0.17	0.18	0.32	1.31	3.74	0.52	1.10	3.45	1.00	–	0.23	0.84
**Nuclear DNA data**															
*AP3*	GTR+Γ	3206.5	0.31	0.16	0.19	0.35	1.02	2.30	0.47	1.57	1.98	1.00	–	0.00	1.60
*phyA*	HKY+I	3293.3	0.27	0.21	0.26	0.27	–	–	–	–	–	–	1.55	0.76	–
Combined data	GTR+Γ	6548.0	0.29	0.19	0.22	0.31	0.88	2.53	0.50	1.44	2.11	1.00	–	0.00	0.78
**Combined chloroplast and nuclear data**															
	GTR+Γ+I	54329.9	0.32	0.17	0.18	0.32	1.31	3.60	0.53	1.10	3.33	1.00	–	0.22	0.84

Substitution models: GTR, General-time-reversible model [45]; HKY, Hasegawa-Kishino-Yano model [46] I, proportion of invariable sites; Γ, among site rate heterogeneity modeled with a gamma distribution.

Support for individual clades was assessed using bootstrap support (BS) in the MP and ML analyses, and posterior probabilities (PP) in the BI analyses. Nodes with BS values of 50–74% were regarded as weakly supported, 75–84% as moderately supported, and 85–100% as strongly supported. Nodes with PP values ≥ 0.95 were considered as strongly supported, and < 0.95 not supported [47].

### Assessing dataset congruence

We assessed the occurrence of ‘hard’ phylogenetic incongruence between the cpDNA and nDNA datasets: ‘hard’ incongruence refers to the presence of strongly supported conflict among datasets [48], which indicates potential hybridization, incomplete lineage sorting or gene duplication. We visually inspected the topologies to determine the presence of conflicting clades that were supported by BS values > 70% or PP values > 0.95 [49].

We furthermore applied the incongruence length difference (ILD) test [50], implemented in PAUP* 4.0b10 [36], with 100 replicates of random stepwise sequence addition and tree bisection-reconnection (TBR) branch swapping. Constant characters were excluded from the dataset to avoid overestimating the level of incongruence [51,52]. Pairwise comparisons among the six regions and the combined dataset as a whole were conducted to assess congruence among gene regions and between the cpDNA and nDNA datasets. It has been suggested that a threshold of p < 0.05 is too conservative for the ILD test [50,53], increasing the probability of type I errors [48]; the null hypothesis of congruence is therefore rejected here at p < 0.01.

### Morphological character evolution

As the analysis primarily focused on *Disepalum* and tribe Annoneae, phylogenetically more distant outgroups with incomplete morphological information were excluded. The final dataset contained 14 taxa, including eight *Disepalum* species, and a single representative species from six other genera in the Annoneae (including *Annona*, *Anonidium*, *Asimina*, *Diclinanona*, *Goniothalamus* and *Neostenanthera*).

Nine morphological characters of potential taxonomic and functional significance for *Disepalum s*.*l*. were selected (Table 3). Character states were categorized with reference to previous morphological studies [4,12,15,17,54,55,56,57,58,59] and novel observations from living and herbarium collections (A, BRUN, HKU, K, KEP, L, NY and S herbaria). For *Disepalum* species, the character states were scored for each species. For the single representatives of other genera in Annoneae, scoring of character states depended on whether the character was monomorphic or polymorphic across all species of the respective genera: for polymorphic character state distributions, either the character state inferred to be ancestral within the genus in previous studies was scored [15,59]; or, in the absence of densely sampled studies providing character state reconstructions, the character state was scored as unknown.

**Table 3:** Matrix of morphological character states used for character mapping.

Taxon	Characters								
	1	2	3	4	5	6	7	8	9
*Anonidium* spp.	0	0	0	1	?	1	0	0	0
*Neostenanthera* spp.	0	0	0	1	0	1	0	1	0
*Goniothalamus* spp.	0	0	0	1	1	0	0	0	0
*Diclinanona* spp.	0	0	0	1	1	1	0	0	0
*Annona* spp.	0	?	?	1	?	1	?	0	0
*Asimina* spp.	0	0	0	?	0	0	0	0	0
*Disepalum petelotii*	0	0	0	1	1	1	1	1	0
*Disepalum plagioneurum*	0	0	0	1	1	1	1	1	0
*Disepalum pulchrum*	0	0	0	1	1	1	1	1	0
*Disepalum platypetalum*	1	0, 1	1	0	0	1	1	1	1
*Disepalum anomalum*	1	1	1	0	0	1	1	1	1
*Disepalum longipes*	1	1	1	0	0	1	1	1	1
*Disepalum aciculare*	1	1	1	0	0	1	1	1	1
*Disepalum coronatum*	1	1	1	0	0	1	1	1	1

Morphological characters: (1) Typical number of sepals per flower: 0 = three; 1 = two. (2) Fusion of petals: 0 = free; 1 = fused. (3) Number of petal whorls per flower: 0 = two; 1 = one. (4) Presence of distinct pigmented region at base of petals: 0 = absent; 1 = present. (5) Mean length of stamens: 0 = < 2 mm; 1 = > 2 mm. (6) Mean number of carpels per flower: 0 = < 10; 1 = > 15. (7) Pollen unit: 0 = monad; 1 = tetrad; 2 = octad. (8) Presence of carpophores: 0 = present; 1 = absent. (9) Persistence of perianth in fruit: 0 = not persistent; 1 = persistent.? = missing data.

A total of 75 common characters regarding floral, fruit and pollen morphologies were investigated. For each character, the phylogenetic signal was evaluated using Pagel’s λ [60] with function phylosig (Package phytools [61]) and fitDiscrete (Package Geiger 3.1 [62]) in R [63], a method described in Tang et al. [28] for discrete characters. For Pagel’s λ, a value of 1 indicates that the character state distribution at the terminals can be explained by phylogenetic patterns, whereas a value of 0 indicates a lack of phylogenetic signal [60]. Only characters with detectable phylogenetic signal (p-value < 0.05) and < 50% missing data were chosen for subsequent ancestral state optimization. Definitions of characters and character states and the resultant data matrix are given in Table 3.

Ancestral character state reconstructions were performed using parsimony and likelihood approaches in Mesquite 2.7.4 [64]. The post-burn-in trees from the Bayesian phylogenetic reconstruction using the combined chloroplast and nuclear DNA sequence data and six data partitions were used as input tree files. The results were mapped on the Bayesian 50% majority-rule consensus tree.

Parsimony ancestral state optimizations were performed with the ‘Trace over trees’ option to account for phylogenetic uncertainty. The results were summarized using the ‘Count trees with uniquely best states’ option and mapped on the Bayesian 50% majority-rule consensus tree. A reconstruction is regarded as equivocal when there are two or more equally parsimonious states inferred at a particular node. In the ML approach, the character state for each ancestral node was reconstructed using the Mk1 model (Markov k-state 1 parameter model; [65]), which specifies an equal probability of any state change and considers the rate of change as the only parameter. The ‘Trace over trees’ option was selected to account for phylogenetic uncertainty, and the results were summarized using the ‘Average frequencies across trees’ option (which calculates the average likelihood of each state at each node across all of the trees possessing that particular node). Since the likelihood approach in Mesquite is not applicable for polymorphic characters, ancestral state reconstructions for character 2 (fusion of petals; Table 3) were performed using only the parsimony approach.

Bayesian ancestral state optimizations were performed using BayesTraits 2.0 [66]. The MCMC mode and the ‘multistate’ model of evolution were selected and the reverse-jump (RJ) hyperprior approach [67] with uniform interval from 0 to 30 used. The ‘addMRCA’ command was applied to calculate the posterior distribution of ancestral character states of each node. A total of 1 billion iterations were run, with sampling every 1000^th^ iteration and 25% burn-in discarded. The ‘ratedev’ parameter for each character was adjusted in order to optimize the acceptance rate (20–40%). Performance of the analyses was evaluated in Tracer 1.5 [42] to ensure adequate effective sample sizes (ESS) for each parameter (ESS > 200) were reached.

### Nomenclature

The electronic version of this article in Portable Document Format (PDF) in a work with an ISSN or ISBN will represent a published work according to the *International Code of Nomenclature for algae*, *fungi*, *and plants*, and hence the new names contained in the electronic publication of a PLOS ONE article are effectively published under that Code from the electronic edition alone, so there is no longer any need to provide printed copies.

In addition, new names contained in this work have been submitted to IPNI, from where they will be made available to the Global Names Index. The IPNI LSIDs can be resolved and the associated information viewed through any standard web browser by appending the LSID contained in this publication to the prefix http://ipni.org/. The online version of this work is archived and available from the following digital repositories: PubMed Central and LOCKSS.

## Results

The results of the ILD tests showed that there was no significant incongruence between pairwise datasets among the six gene regions (all p-values > 0.01; Table 4) and the cpDNA and nDNA partitions (p-value: 1.00).

**Table 4:** The p-values of pairwise incongruence length difference (ILD) tests among the six chloroplast and nuclear DNA markers.

	*matK*	*trnL-F*	*ndhF*	*ycf1*	*AP3*
*trnL-F*	0.30	–			
*ndhF*	1.00	0.53	–		
*ycf1*	0.90	0.51	0.47	–	
*AP3*	1.00	1.00	1.00	1.00	–
*phyA*	1.00	1.00	1.00	0.81	1.00

Significant incongruence at p < 0.01

For the BI analysis, partitioning generally improved the mean—ln*L* values in all analyses of the chloroplast sequence data (mean—ln*L*_unpartitioned_ = 23,902; mean—ln*L*_partitioned_ = 23,742), nuclear sequence data (mean—ln*L*_unpartitioned_ = 3,263; mean—ln*L*_partitioned_ = 3,254) and combined data (mean—ln*L*_unpartitioned_ = 26,977; mean—ln*L*_2-partitioned_ = 26,952; mean—ln*L*_6-partitioned_ = 26,781). Bayes factor comparison between the unpartitioned and partitioned models also indicated that the partitioned model provided better explanations of the data: chloroplast data: 2ln Bayes factor (partitioned over unpartitioned) = 317; nuclear: 2ln Bayes factor (partitioned over unpartitioned) = 15; combined: 2ln Bayes factor (6-partitioned over 2-partitioned) = 338; 2ln Bayes factor (6-partitioned over unpartitioned) = 387). All results presented here are therefore based on analyses of the datasets partitioned according to DNA region identity.

**Fig 2:**
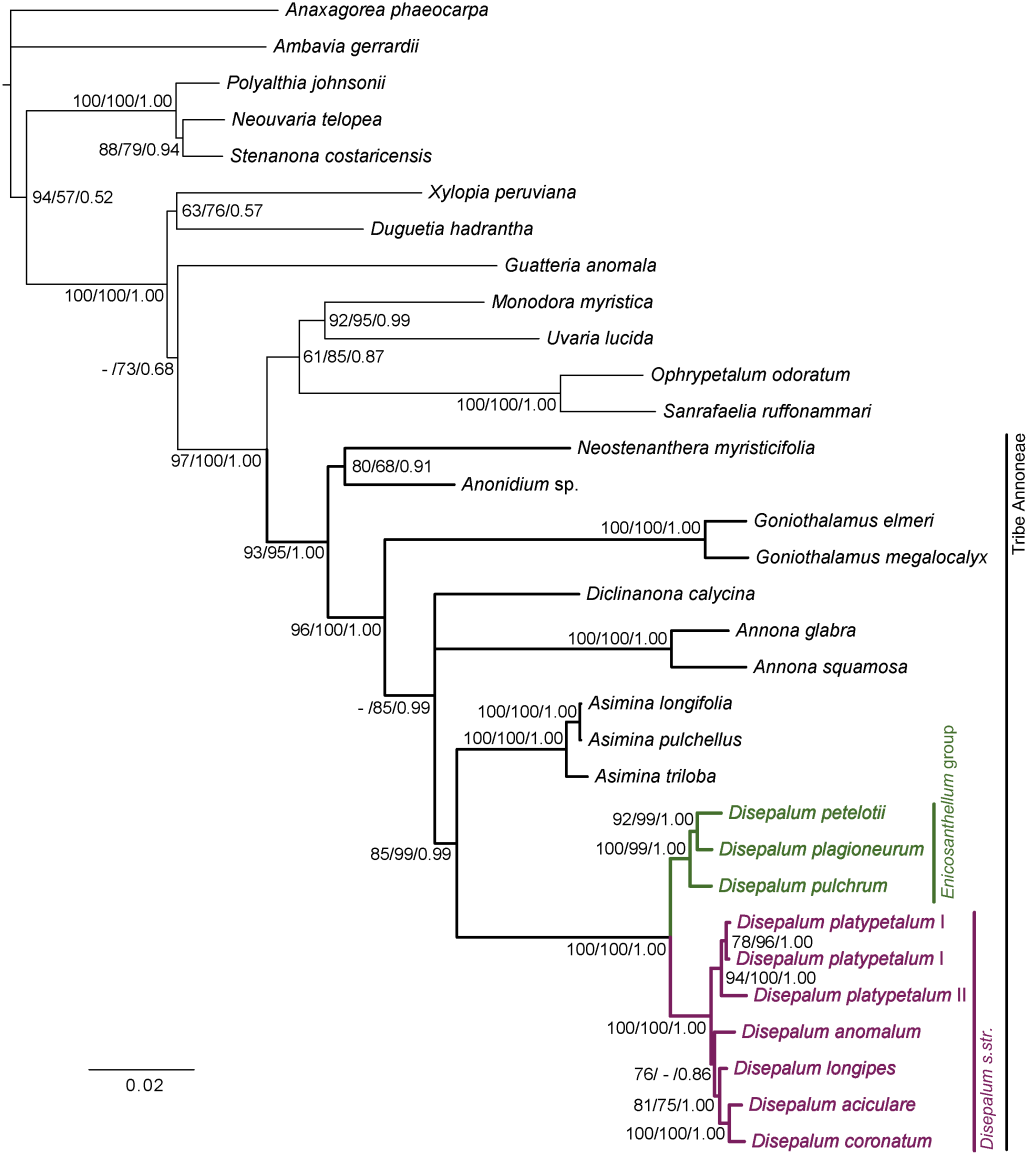
Bayesian 50% majority-rule consensus tree under partitioned model based on combined cpDNA (*matK*, *trnL-F*, *ndhF* and *ycf1*) and nDNA (*AP3* and *phyA*) DNA sequence data. Numbers at nodes indicate MP, ML bootstrap values (> 50%) and Bayesian posterior probabilities (> 95%). Bootstrap values < 50% are indicated by ‘-’. Scale bar: 0.02 substitutions per site.

The MP, ML and BI tree topologies are highly congruent (Fig 2) and are relatively well resolved and strongly supported. The topology shows that the tribe Annoneae is monophyletic (MP BS = 93%; ML BS = 95%; PP = 1.00). All eight *Disepalum* species sampled in this study also form a monophyletic group (MP BS = 100%; ML BS = 100%; PP = 1.00), comprising two unambiguous subclades representing *Disepalum s*.*str*. (MP BS = 100%; ML BS = 100%; PP = 1.00) and *Enicosanthellum* (MP BS = 100%; ML BS = 99%; PP = 1.00). The results show that specimens representing the two distinct floral types of *D*. *platypetalum* consistently form a strongly supported clade (MP BS = 94%; ML BS = 100%; PP = 1.00), which is an early-divergent lineage within the *Disepalum s*.*str*. clade, although its sister clade only receives moderate support at best (MP BS = 76%; ML BS < 50%; PP = 0.86).

**Fig 3:**
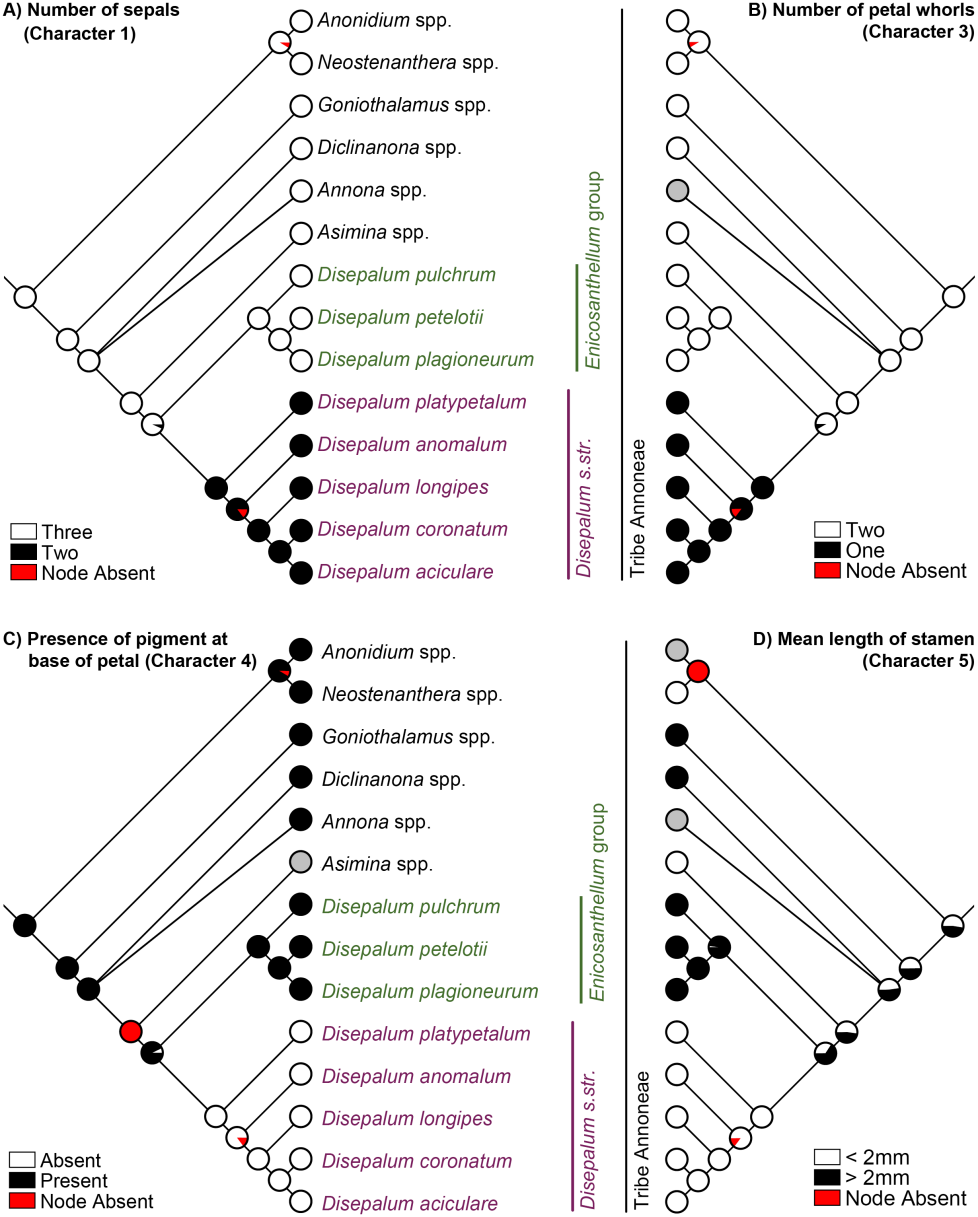
Likelihood ancestral character state estimation for *Disepalum* and the tribe Annoneae across 90,000 Bayesian input trees, mapped on the Bayesian majority-rule consensus tree under a partitioned model based on combined cpDNA (*matK*, *trnL-F*, *ndhF* and *ycf1*) and nDNA (*AP3* and *phyA*) sequence data. (A) Number of sepals (character 1). (B) Number of petal whorls (character 3). (C) Presence of pigment at base of petals (character 4). (D) Mean length of stamens (character 5). Pie charts at each node show the percentage of node absence in the input trees and the average likelihood received by each state across all input trees possessing that node. Grey circles indicate unknown character states.

**Fig 4:**
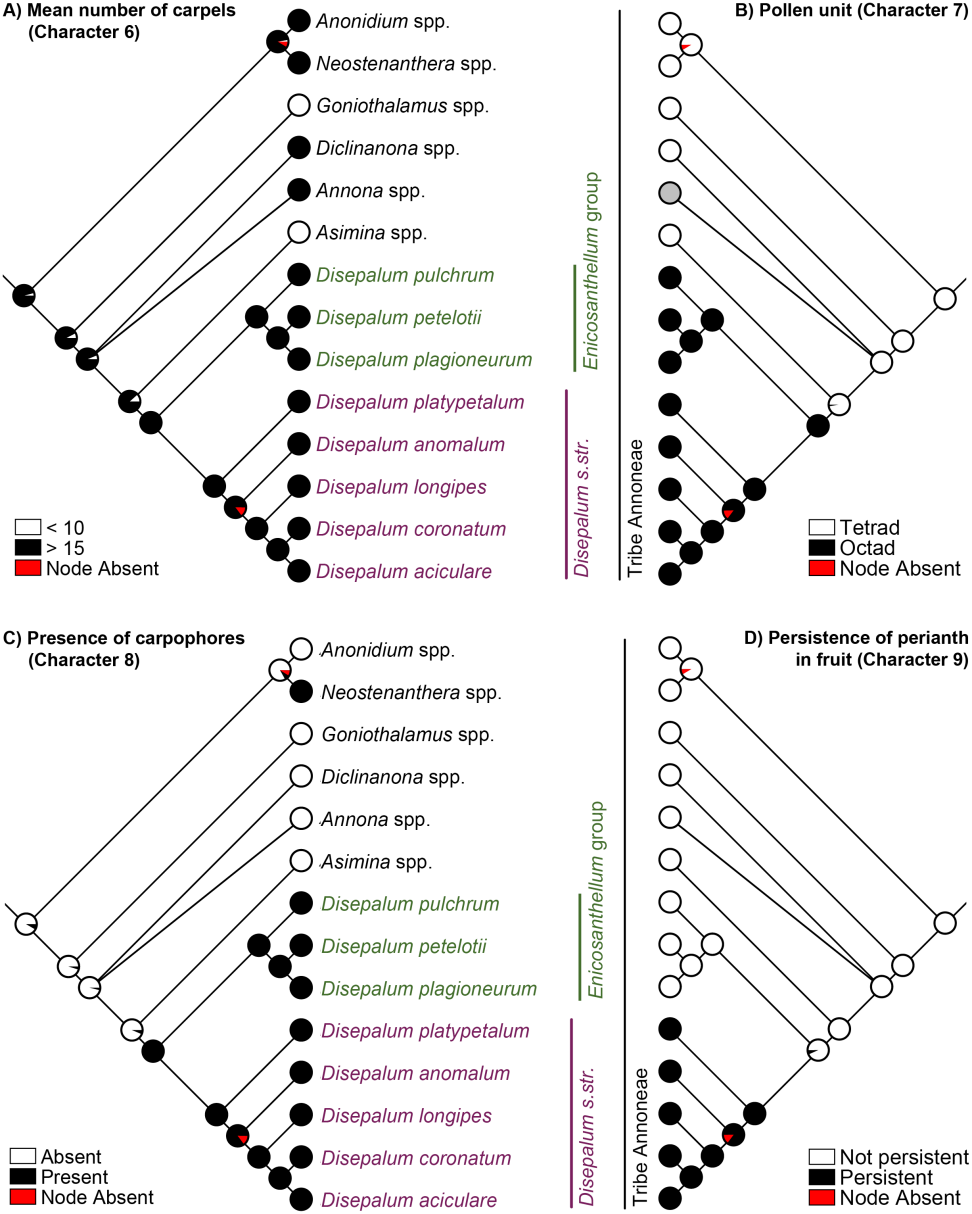
Likelihood ancestral character state estimation for *Disepalum* and the tribe Annoneae across 90,000 Bayesian input trees, mapped on the Bayesian majority-rule consensus tree under a partitioned model based on combined cpDNA (*matK*, *trnL-F*, *ndhF* and *ycf1*) and nDNA (*AP3* and *phyA*) sequence data. (A) Mean number of carpels (character 6). (B) Pollen unit (character 7). (C) Presence of carpophores (character 8). (D) Persistence of perianth in fruit (character 9). Pie charts at each node show the percentage of node absence in the input trees and the average likelihood received by each state across all input trees possessing that node. Grey circles indicate unknown character states.

Strong phylogenetic signal was detected for all selected characters (Pagel’s λ = 1, p < 0.05; Table 5). The results of the parsimony, likelihood and Bayesian analyses of evolutionary patterns in selected flower and fruit morphological characters are highly congruent, with one exception: the mapping of mean stamen length (character 5) at the ancestral node of the tribe Annoneae. The evolutionary patterns of all characters using the likelihood approach are presented in Figs 3 and 4, the results of the parsimony reconstructions and likelihood estimations are presented in Table 5, and the results of the Bayesian estimations in Table 6.

**Table 5:** Evaluation of phylogenetic signal of the nine selected morphological characters using Pagel’s λ, resultant ancestral character state by parsimony optimization and average proportional likelihoods by likelihood ancestral character reconstruction based on Bayesian trees generated under partitioned model using combined data of four cpDNA (*matK*, *trnL-F*, *ndhF* and *ycf1*) and two nDNA (*AP3* and *phyA*) markers.

Character	λ	P-value	Character states	Tribe Annoneae	*Anonidium- Neostenanthera*	*Goniothalamus- Annona*	*Annona- Asimina*	*Asimina- Disepalum*	*Disepalum*	Subgen. *Enicosanthellum*	Subgen. *Disepalum*
1	1	0.0040	0	0.9990*	0.9994*	0.9995*	0.9999*	0.9991*	0.9338*	0.9996*	0.0011
			1	0.0010	0.0006	0.0005	0.0001	0.0009	0.0662	0.0004	0.9989*
2	1	0.0169	0	*	*	*	*	*	*	*	
			1								*
3	1	0.0053	0	0.9981*	0.9987*	0.9991*	0.9993*	0.9983*	0.9173*	0.9994*	0.0013
			1	0.0019	0.0013	0.0009	0.0007	0.0017	0.0827	0.0006	0.9987*
4	1	0.0053	0	0.0015	0.0009	0.0010	0.0009	–	0.0747	0.0005	0.9988*
			1	0.9985*	0.9991*	0.9990*	0.9991*	– *	0.9253*	0.9995*	0.0012
5	1	0.0244	0	0.5264*	– *	0.4982*	0.4851	0.5300*	0.3428*	0.0207	0.9921*
			1	0.4736	–	0.5018	0.5149	0.4700	0.6572	0.9793*	0.0079
6	1	0.0396	0	0.0438	0.0318	0.0759	0.0642	0.1200	0.0013	0	0
			1	0.9562*	0.9682*	0.9241*	0.9358*	0.8800*	0.9987*	1*	1*
7	1	0.0009	0	0.9981*	0.9988*	0.9982*	0.9962*	0.9863*	0.0029	0	0
			1	0.0019	0.0012	0.0018	0.0038	0.0137	0.9971*	1*	1*
8	1	0.0034	0	0.9178*	0.9095*	0.9548*	0.9724*	0.9504*	0.0061	0.0001	0
			1	0.0822	0.0905	0.0452	0.0276	0.0496	0.9939*	0.9999*	1*
9	1	0.0040	0	0.9990*	0.9994*	0.9995*	0.9999*	0.9991*	0.9338*	0.9996*	0.0011
			1	0.0010	0.0006	0.0005	0.0001	0.0009	0.0662	0.0004	0.9989*

Only parsimony ancestral character reconstruction was performed for character B due to polymorphism. Absent nodes and equivocal results in the likelihood ancestral character reconstruction are indicated by ‘–’.

The resultant ancestral character state determined by maximum parsimony is represented by * (MP = 1.00 at each node for all characters). Numbers in bold font indicate shifts of character state (i.e., synapomorphies). For definitions of characters and character states see Table 3.

**Table 6:** Bayesian ancestral character reconstruction across 90,000 Bayesian input trees generated under partitioned model using combined data of four cpDNA (*matK*, *trnL-F*, *ndhF* and *ycf1*) and two nDNA (*AP3* and *phyA*) markers.

Character	Character states	Tribe Annoneae	*Anonidium- Neostenanthera*	*Goniothalamus- Annona*	*Annona- Asimina*	*Asimina- Disepalum*	*Disepalum*	Subgen. *Enicosanthellum*	Subgen. *Disepalum*
1	0	0.9468	0.9300	0.9201	0.9425	0.8825	0.7118	0.9983	0.0007
	1	0.0532	0.0700	0.0799	0.0575	0.1175	0.2882	0.0017	**0.9993**
2	0	0.9282	0.9091	0.8918	0.9138	0.8625	0.7252	0.9974	0.0090
	1	0.0718	0.0909	0.1082	0.0862	0.1375	0.2748	0.0026	**0.9910**
3	0	0.9321	0.9131	0.8959	0.9171	0.8641	0.7110	0.9977	0.0009
	1	0.0679	0.0869	0.1041	0.0829	0.1359	0.2890	0.0023	**0.9991**
4	0	0.0804	0.0904	0.1248	0.1125	0.2233	0.2962	0.0025	**0.9990**
	1	0.9196	0.9096	0.8752	0.8875	0.7767	0.7038	0.9975	0.0010
5	0	0.6325	0.6995	0.4518	0.4829	0.6437	0.3614	0.0086	**0.9965**
	1	0.3675	0.3005	0.5482	0.5171	0.3563	0.6386	0.9914	0.0035
6	0	0.1489	0.1127	0.3295	0.2235	0.5417	0.0094	0.0025	0.0010
	1	0.8511	0.8873	0.6705	0.7765	0.4583	0.9906	0.9975	0.9990
7	0	0.9490	0.9384	0.9003	0.8943	0.6796	0.0046	0.0012	0.0005
	1	0.0510	0.0616	0.0997	0.1057	0.3204	**0.9954**	**0.9988**	**0.9995**
8	0	0.6578	0.4057	0.7839	0.8071	0.6081	0.0112	0.0030	0.0012
	1	0.3422	0.5943	0.2161	0.1929	0.3919	**0.9888**	**0.9970**	**0.9988**
9	0	0.9467	0.9299	0.9200	0.9424	0.8825	0.7118	0.9983	0.0007
	1	0.0533	0.0701	0.0800	0.0576	0.1175	0.2882	0.0017	**0.9993**

Numbers in bold font indicate shifts of character state (i.e., synapomorphies). For definitions of characters and character states see Table 3.

## Discussion

### Taxonomic delimitation

The monophyletic status of *Disepalum s*.*l*., *Disepalum s*.*str*. and *Enicosanthellum* are all confirmed by the MP, ML and BI analyses (Fig 2), supporting two alternative interpretations of generic delimitation: either a broad delimitation of *Disepalum*, inclusive of *Enicosanthellum*; or a narrower delimitation of *Disepalum s*.*str*., following segregation of its sister genus, *Enicosanthellum*.

The formation of pollen octads (Fig 4B: character 7) and presence of carpophores (Fig 4C: character 8) are synapomorphic for *Disepalum s*.*l*. *Disepalum s*.*str*. furthermore possesses six floral character states that are likely to be synapomorphic, including: reduction of the calyx to only two sepals (Fig 3A: character 1); fusion of petals (Table 5: character 2); loss of one of the two petal whorls (Fig 3B: character 3); loss of the pigment at the petal base (Fig 3C: character 4); reduction in stamen length to less than 2 mm (Fig 3D: character 5), and persistence of the perianth in fruit (Fig 4D: character 9). It is possible that characters 1–3 may not be independent, however: the occurrence of specific character states may be directly or indirectly channeled by common genetic mutations and developmental paths. This will be discussed in greater detail below, under ‘Perianth morphology’.

There are no obvious synapomorphies for *Enicosanthellum*, although several diagnostic plesiomorphic character states separate this lineage from *Disepalum s*.*str*., including the trimerous calyx (Fig 3A: character 1), free petals in two distinct whorls (Table 5: character 2; Fig 3B: character 3), pigmented base of petals (Fig 3C: character 4), stamens that are longer than 2 mm (Fig 3D: character 5), and absence of the perianth in fruit (Fig 4D: character 9). The lack of morphological synapomorphies for the *Enicosanthellum* clade is a cogent argument against its recognition as a distinct genus, and we therefore support the broader delimitation of *Disepalum* previously adopted by Johnson [2].

The results of the ILD test suggest that the cpDNA and nDNA datasets are congruent. Although this evidence, together with the lack of hard incongruence between the chloroplast and nuclear phylogenies (S4 and S5 Files), suggests that *D*. *platypetalum* is unlikely to be of hybrid origin, it is significant that the node between *D*. *platypetalum* and the rest of the *Disepalum s*.*str*. clade in the nDNA phylogeny has inadequate support and hence does not rule out alternative phylogenetic positions for the species. Additional nDNA data is necessary to clarify the phylogenetic relationship between *D*. *platypetalum* and the *Enicosanthellum* group and hence determine whether *D*. *platypetalum* belongs to a lineage that originated by hybridization between the two groups.

Although we recommend adopting a broader delimitation of *Disepalum*, inclusive of *Enicosanthellum*, we nevertheless propose that the two well-supported clades within the genus (*Disepalum s*.*str*. and the *Enicosanthellum* clade) should be recognized as subgenera. These clades are morphologically very distinct, and although the diagnostic morphological character states of subgen. *Enicosanthellum* are symplesiomorphies, this is unlikely to be problematic since a non-principal rank is applied. The descriptions of the two subgenera and the nomenclatural validation of subgen. *Enicosanthellum* are as follows:

### *Disepalum* Hook. f. subgen. *Disepalum*

Type species: *Disepalum anomalum* Hook. f.

Inflorescences leaf-opposed or sometimes terminal, 1 or 2-flowered; pedicels 1.4–9 cm. Floral torus 4–10 mm in diameter. Sepals 2 per flower, 5–12 mm long, 5–11 mm wide, orbicular to ovate. Petals connate (sometimes both free and connate within the same flower in *D*. *platypetalum*), in one whorl; corolla tube 2–11 mm long; corolla lobes 4–9 per flower, 2–12.5 mm long, 0.5–5.5 mm wide, triangular, linear or spathulate. Stamens 2.2–3.8 mm long. Calyx and corolla persistent in fruit.

Included species: *D*. *aciculare* D.M. Johnson, *D*. *acuminatissimum* Boerl. & Koord., *D*. *anomalum* Hook. f., *D*. *coronatum* Becc., *D*. *longipes* King, and *D*. *platypetalum* Merr.

### *Disepalum* Hook. f. subgen. *Enicosanthellum* (Bân) P.S. Li, D.C. Thomas & R.M.K. Saunders, subgen. nov.

[urn:lsid:ipni.org:names:77150817–1]

Basionym: *Enicosanthellum* Bân, Bot. Žurn. (Moscow & Leningrad) 60: 812. 1975.

Type species: *Disepalum petelotii* (Merr.) D.M. Johnson.

Inflorescences terminal, 1-flowered; pedicels 1.2–2 cm. Floral torus 2.5–8 mm in diameter. Sepals 3 per flower, 10–21 mm long, 7.5–17 mm wide, ovate or broadly ovate. Petals free, in 2 whorls of 3, oblong or oblanceolate; outer petals 13–44 mm long, 6.5–25 mm wide; inner petals 13–34 mm long, 7–24 mm wide. Stamens 1–2.1 mm long. Calyx and corolla not persistent in fruit.

Included species: *D*. *petelotii* (Merr.) D.M. Johnson, *D*. *plagioneurum* (Diels) D.M. Johnson, and *D*. *pulchrum* (King) J. Sinclair

### Perianth morphology

A strong correlation is apparent between the three perianth characters mapped, which are all synapomorphic for subgen. *Disepalum*: number of sepals per flower (Fig 3A: character 1); fusion of petals; and number of whorls of petals (Fig 3B: character 3). *Disepalum platypetalum* (Fig 1C) is the only species in the genus that is not fully consistent with this apparent correlation: the flowers of this species are very variable in the number of corolla lobes (indicative of the number of fused petals) and the degree of fusion of petals [2]. All flowers of *D*. *platypetalum* exhibit adnation of the corolla with the receptacle, but either have free or connate petals, sometimes even within the same flower [2]. Our observations of *D*. *platypetalum* (based solely on herbarium material due to difficulties accessing wild populations) suggest that a suture is apparent between contiguous fused petals, indicating that the fusion is likely to be postgenital. This contrasts with other species in subgen. *Disepalum* (e.g., *D*. *anomalum*: Fig 1B), which appear to show congenital fusion of petals, without any evidence of a suture. Since *D*. *platypetalum* is the first lineage to diverge within subgen. *Disepalum* (Fig 2) it is possible that congenital fusion of petals is synapomorphic for the *D*. *anomalum-longipes-aciculare-coronatum* clade.

Saunders [4] suggested that the reduction of petal whorls in subgen. *Disepalum* involved compression of inner and outer whorls into one single whorl—a transition that is often associated with petal connation in the Annonaceae (e.g., *Isolona* and *Monodora*: [68]). The genetic control of floral morphological changes within *Disepalum* is largely unknown, but substantial floral changes in the Annonaceae have been hypothesized to be a consequence of a disruption of the genetic control of organ identity during floral development [4]. The ABCDE model has been proposed to explain the genetic control of floral organ identity in eudicots, suggesting that there are at least five classes of homeotic genes (essentially MADS-box genes) determining floral organ identity [69,70,71,72]. This model is also likely to be applicable to the Annonaceae, with convergent evolution of the homeotic genetic control mechanism tentatively identified in the family (*Asimina longifolia*: [73]). The hypothesis that petal fusion in subgen. *Disepalum* may have resulted from disruption of the expression of MADS-box genes is unlikely, however, since these homeotic genes are mainly responsible for determining organ identity. The disruption of MADS-box gene expression might increase the likelihood of petal fusion, however: the putative association between compression of petal whorls and fusion of petals [4], suggests that compression is a possible prerequisite for fusion as it results in the close spatial arrangement of petal primordia and their almost simultaneous development [74,75].

The genetic control of floral organ fusion has been investigated in various angiosperm lineages (e.g., *Arabidopsis*: [76]; *Petunia*: [77]). In *Petunia*, the *MAEWEST* (*MAW*) and *CHORIPETALA SUZANNE* (*CHSU*) genes have overlapping roles in controlling fusion of carpels and petals as well as the lateral growth of leaf laminas: expression failure of these genes would cause differing levels of disruption in fusion of the organs [77]. Lateral development of organs is severely reduced in *maw chsu* double mutants, whilst a degree of organ fusion was observed in either one of the two mutants [77]. The Annonaceae genome has not been well studied and the genetic control of each floral organ is largely unknown. Nevertheless, the *Petunia* example might provide some clues in understanding the transition from free to connate petals within *Disepalum*, and also the origin of intermediate floral morphology in *Disepalum platypetalum*, suggesting that various degree of petal fusion in subgen. *Disepalum* could be the consequence of expression failure of one or more of the relevant genes.

The development of a single whorl of connate petals in subgen. *Disepalum* is correlated with the loss of a functional pollination chamber: the floral reproductive organs in subgen. *Enicosanthellum* (e.g, *D*. *pulchrum*: Fig 1A) are enclosed within a chamber formed by the petals throughout floral receptivity, whereas those of subgen. *Disepalum* (e.g., *D*. *anomalum* and *D*. *platypetalum*: Fig 1B and 1C) are always fully exposed. Although pollination chambers are widespread in the Annonaceae, they are structurally very diverse (derived from the inner or outer petals or a combination of both whorls, with different parts of the petals in mutual contact and with differing degrees of connation), and hence are not homologous in many cases [4]. Pollination chambers nevertheless appear to perform a very similar function throughout the family, providing pollinators (generally beetles) with a protected environment and often helping to maintain micro-environments, including elevated internal temperatures that encourage beetle mobility [4,78].

The pollination ecology of *Disepalum* species is largely unknown, although flowers of *D*. *pulchrum* (subgen. *Enicosanthellum*) have been reported to be visited by beetles [2]. The floral morphology of species in subgen. *Enicosanthellum* is consistent with cantharophily, and small-beetle pollination has furthermore been inferred as the ancestral pollination system in the tribe Annoneae as a whole [78]. The evolutionary loss of the pollination chamber in subgen. *Disepalum*, associated with changes in the number, arrangement and fusion of petals, would have had a major impact on pollination ecology: although beetle pollination is known to occur in other Annonaceae genera that lack a pollination chamber (e.g., *Uvaria*: [78] and references therein), loss of chambers is typically associated with less specialized pollination systems, in which a broader array of pollinators are attracted. This is perhaps most obvious in *Uvaria*, in which open-flowered species have been reported to be pollinated by small beetles [79,80,81], cockroaches [82], drosophilid flies [82], and meliponine bees [83]. In this context, it is significant that flowers of *D*. *anomalum* (subgen. *Disepalum*) have been observed to be visited by meliponine bees (P.S. Li, unpubl. data: Fig 1G). This suggests that the changes in floral morphology apparent in *Disepalum* may be correlated with a change in pollination system, although more detailed comparative field studies of the pollination ecology of representative species of the two subgenera are required to assess the impact of the evolutionary loss of pollination chambers.

### Pollen aggregation and numerous carpels

The pollen of the majority of Annonaceae species is dispersed as solitary grains (monads) at maturity. This has been inferred as the plesiomorphic condition for the family, with pollen aggregation derived in several phylogenetically disparate lineages across three of the four subfamilies (Ambavioideae, Malmeoideae and Annonoideae) [15]. Compound pollen is particularly widespread in subfam. Annonoideae, in which it is synapomorphic for the tribes Annoneae, Bocageeae and Monodoreae [15]. The results of the character mapping (Fig 4B: character 7) indicate that tetrad pollen is the ancestral condition within the tribe Annoneae, with pollen octads (Fig 1D) synapomorphic for *Disepalum s*.*l*.

Pollen tetrads form as a result of the four microspores derived from each microsporocyte failing to dissociate; pollen octads follow a similar developmental sequence, but are derived from twin sporocytes that develop in synchrony [84]. The development of pollen tetrads in *Annona* (tribe Annoneae [85,86,87]) and octads in *Cymbopetalum* (tribe Bocageeae [84]) exhibit a remarkable developmental sequence, in which the thin proximal area of the pollen wall (towards the centre of the compound pollen) initially develops distally, and alters position due to rotation of the developing microspores. It is unclear whether a similar developmental pattern is evident in *Disepalum*, however, as the closely related genus *Asimina* does not exhibit this phenomenon [88,89,90].

A strong correlation exists in the Annonaceae between the occurrence of compound pollen and the formation of septate anthers [12,86,91,92]). Although most species in the Annonaceae have aseptate anthers, lacking any internal division within the thecae during pollen development, others have thecae that are at least temporarily compartmentalized into internal chambers with a single compound pollen unit in each chamber; in these cases the septa are either tapetal, degenerating prior to dehiscence, or else are parenchymatous, retained throughout development and are evident in the thecae at dehiscence [86]. Thecae in *Disepalum* are clearly septate (Fig 1H), with septa that are tapetal as they are not evident in mature anthers (Fig 1I); this observation is consistent with data available for the closely related genera *Asimina* and *Annona*, although *Goniothalamus* (also from tribe Annoneae) differs in possessing parenchymatous septa [86]. One of the hypotheses proposed to explain the correlation between compound pollen and anther septation is that the septa enable greater contact between the tapetum and the developing pollen, meeting the greater nutritive demands of large pollen aggregates [86,93,94,95].

Pollen aggregation is likely to be selectively advantageous under unfavorable conditions in which pollinator visits are infrequent, since a single visit could potentially transfer multiple pollen grains [96]. The benefits of the transfer of pollen aggregates would only be achieved, however, in flowers in which there are multiple ovules available for fertilization. Although there are generally only two (rarely one or three) ovules per carpel in *Disepalum*, there are numerous carpels in each flower, with up to 200 in several species [2]. As with most Annonaceae, however, *Disepalum* flowers are apocarpous, and so it is hypothesized that pollen tubes are likely to be able to grow between contiguous carpels: this extragynoecial growth of pollen tubes has been reported in other apocarpous angiosperms [97,98,99]. *Disepalum* flowers, as with many Annonaceae, develop copious stigmatic exudate during the pistillate receptive phase, and it is suggested that this exudate may act as an extragynoecial compitum, enabling pollen tubes to potentially reach any carpel.

### Fruit morphology

Fruit morphology is very diverse in the Annonaceae [14], and is associated with different frugivores and seed dispersal patterns. In most species the fruits are apocarpous, with carpels that remain as separate ‘monocarps’ in the mature fruit: these monocarps are either sessile, in which case the entire fruit is typically removed and consumed by the frugivore as a single unit; or else the monocarps are borne on stalks and are separated from the rest of the fruit by the frugivore. Monocarp stalks are often observed in Annonaceae fruits with smaller monocarps that are bird-dispersed, although there are also many examples of larger monocarps borne on stalks. The monocarp stalks are generally carpellate in origin, developing as an extension of the base of the monocarp, and consequently with a basal articulation where it separates from the rest of the fruit; in these cases the monocarp stalk is referred to as the ‘stipe’. In a small number of genera, however, including *Disepalum* (Fig 1E and 1F) and *Neostenanthera* in tribe Annoneae, the monocarp stalk develops as an extension of the receptacle, and hence the articulation is distal [2,14]; in these cases the stalk is known as the ‘carpophore’. The function of the stipe and carpophore appears to be identical: to assist in the separation of individual monocarps from the remainder of the fruit by the frugivore. The convergent evolution of carpophores in *Disepalum* and *Neostenanthera* may have arisen by independent evolution from ancestors that lacked monocarp stalks: these two genera are phylogenetically sister to genera that largely lack stipes (Fig 4C: character 8): *Asimina*, which is sister to *Disepalum*, generally has sessile monocarps (although some species have short stipes [55]); and *Anonidium*, which is sister to *Neostenanthera*, has syncarpous fruits [14].

The carpophores and monocarps are often of contrasting color in *Disepalum* (Fig 1E), possibly associated with the different developmental origin of the two organs and the clearly demarcated boundary between the two. Although there are no reports of frugivory in *Disepalum*, it is likely that birds consume the monocarps. Fruit color has been recognized as a key character as it determines the likelihood of fruits being noticed and consumed, and hence the likelihood of seed dispersal [100]. Fruits consumed by birds are mostly red and black to human perception [100,101], although the color spectrum that can be perceived by birds extends into ultraviolet [101]. Contrasting colors are also likely to play a key role in attracting bird frugivores [100,101]: in a study evaluating the color contrast between fruits and natural backgrounds (including unripe fruits and other vegetative structures) across 43 Neotropical species [101], it was observed that birds displayed a preference for contrasting colors over unicolored fruits. The contrasting colors of the carpophore and the monocarp are therefore likely to have a selective advantage in the effective attraction of birds as seed dispersal agents.

## Supporting Information

S1 File Voucher information and GenBank accession numbers for samples used in this study. (DOCX)

S2 File List of primers used for amplification and sequencing of the four cpDNA (*matK*, *trnL-F*, *ndhF* and *ycf1*) and two nDNA (*AP3* and *phyA*) regions. (DOCX)

S3 File List of protocols used to amplify the four cpDNA (*matK*, *trnL-F*, *ndhF* and *ycf1*) and two nDNA (*AP3* and *phyA*) regions. (DOCX)

S4 File Bayesian 50% majority-rule consensus tree based on combined cpDNA (*matK*, *trnL-F*, *ndhF* and *ycf1*) sequence data partitioned according to DNA region identity. Numbers at nodes indicate MP, ML bootstrap values (> 50%) and Bayesian posterior probabilities (> 95%). Bootstrap values < 50% are indicated by ‘-’. Scale bar: 0.02 substitutions per site.
(TIF)

S5 File Bayesian 50% majority-rule consensus tree based on combined nDNA (*AP3* and *phyA*) sequence data partitioned according to DNA region identity. Numbers at nodes indicate MP, ML bootstrap values (> 50%) and Bayesian posterior probabilities (> 95%). Bootstrap values < 50% are indicated by ‘-’. Scale bar: 0.02 substitutions per site.
(TIF)
