# Supplementary material for: Phylogenetic Reconstruction, Morphological Diversification and Generic Delimitation of Disepalum (Annonaceae)
Source: PLoS One. 2015 Dec 2;10(12):e0143481. doi: 10.1371/journal.pone.0143481 (PMC4668016; doi:10.1371/journal.pone.0143481)
Supplement: S1 File — (DOCX) [file pone.0143481.s001.docx]

**S1 File. Voucher information and GenBank accession numbers for samples used in this study.** Herbarium acronyms for voucher specimens: A (Arnold Arboretum), BRUN (Brunei Forestry Centre), CNS (Australian Tropical Herbarium), FLAS (Florida Museum of Natural History), HKU (The University of Hong Kong), K (Royal Botanic Gardens, Kew), KEP (Forest Research Institute Malaysia), L (Naturalis, Leiden), MISS (University of Mississippi), MO (Missouri Botanical Garden), NY (The New York Botanical Garden), P (Muséum National d’Histoire Naturelle, Paris), S (Swedish Museum of Natural History), U (Naturalis, Leiden), and WAG (Naturalis, Leiden). Newly generated sequences indicated by *.

| Species | Provenance | Voucher | GenBank accession numbers | | | | | |
| --- | --- | --- | --- | --- | --- | --- | --- | --- |
|  |  |  | *matK* | *trnL-F* | *ndhF* | *ycf1* | *AP3* | *phyA* |
| *Ambavia gerrardii* (Baill.) Le Thomas | － | *Sauquet 23* (P) | AY220435 | － | AY218168 | － | － | － |
|  | Madagascar | *Rabevohitra 2035* (MO) | － | JQ513889 | － | － | － | － |
| *Anaxagorea phaeocarpa* Mart. | Ecuador | *Maas et al. 8592* (U) | AY238960 | EF179316 | EF179279 | － | － | － |
| *Annona glabra* L. | USA | *Chatrou 467* (U) | DQ125050 | － | EF179281 | － | － | － |
|  | － | *Abbott 17655* (FLAS) | － | GQ139891 | － | GU937365 | － | － |
| *Annona squamosa* L. | Hong Kong | *Li LPS11* (HKU) | KT452823* | KT452845* | KT452834* | KT452856* | － | － |
| *Anonidium* sp. | Cameroon | *Cheek 7896* (K) | DQ125051 | AY841675 | EF179283 | － | － | － |
| *Asimina angustifolia* A. Gray | － | *Abbott 22368* (FLAS) | GQ139707 | GQ139882 | － | GU937355 | － | － |
|  | USA | *Weerasooriya s.n.* (MISS) | － | － | EF179286 | － | － | － |
| *Asimina pulchellus* Small | － | *Abbott 23697* (FLAS) | GQ139714 | GQ139889 | － | GU937362 | － | － |
| *Asimina triloba* (L.) Dunal | － | *Abbott 23521* (FLAS) | GQ139711 | GQ139886 | － | GU937359 | － | － |
|  | － | *Chatrou et al. 276* (U) | － | － | EF179287 | － | － | － |
| *Diclinanona calycina* *(Diels) R.E.Fr.* | Peru | *Pirie 116* (U) | KC196271 | KC196272 | － | － | － | － |
| *Disepalum anomalum* Hook. f. | Borneo | *Tang TCC36* (BRUN) | KT452819* | KT452841* | KT452830* | KT452852* | KT452807* | KT452860* |
| *Disepalum aciculare* D.M. Johnson | Borneo | *Anderson S13118* (L) | KT452821* | KT452843* | KT452832* | KT452854* | KT452809* | KT452862* |
| *Disepalum coronatum* Becc. | Borneo | *Beaman 8064* (S) | KT452820* | KT452842* | KT452831* | KT452853* | KT452808* | KT452861* |
| *Disepalum longipes* King | Malaysia | *Chin 2165* (L) | KT452822* | KT452844* | KT452833* | KT452855* | － | － |
| *Disepalum plagioneurum* D.M. Johnson | Hainan | *Li LPS10* (HKU) | KT452814* | KT452836* | KT452825* | KT452847* | KT452810* | KT452857* |
| *Disepalum platypetalum* Merr. | Sumatra | *Takeuchi 18733* (A) | KT452816* | KT452838* | KT452827* | KT452849* | KT452807* | KT452859* |
|  | Sumatra | *Nagamasu 3966* (L) | KT452818* | KT452840* | KT452829* | KT452851* | － | － |
|  | Sumatra | *Boeea 11251* (NY) | KT452817* | KT452839* | KT452828* | KT452850* | － | － |
| *Disepalum petelotii* D.M. Johnson | Yunnan | *Feng 11885* (A) | KT452813* | KT452835* | KT452824* | KT452846* | KT452804* | － |
| *Disepalum pulchrum* D.M. Johnson | Malaysia | *Li LPS6* (KEP) | KT452815* | KT452837* | KT452826* | KT452848* | KT452805* | KT452858* |
| *Duguetia hadrantha* (Diels) R.E. Fr. | Peru | *Chatrou 181* (U) | AY740541 | AY740573 | EF179293 | － | － | － |
| *Goniothalamus elmeri* Merr. | Philippines | *Rosario et al. 11-014* (University of Santo Tomas Herbarium) | KM818582 | KM818882 | KM818639 | KM819003 | KT452811* | － |
| *Goniothalamus megalocalyx* I.M. Turner & R.M.K. Saunders | Borneo | *Tang et al. TCC117* (HKU) | KM818600 | KM818885 | KM818645 | KM819007 | KT452812* | － |
| *Guatteria anomala* R.E. Fr. | Mexico | *Ishiki et al. 2233* (U) | AY740913 | AY741011 | EF179298 | － | － | － |
| *Polyalthia johnsonii* (F. Muell.) B. Xue & R.M.K. Saunders | Australia | *Ford AF 3625* (CNS) | JX544826 | JX544801 | JX544840 | JX544819 | － | － |
| *Monodora myristica* (Gaertn.) Dunal | Cultivated | *Chatrou 477* (U) | EU169700 | AY743466 | EU169721 | － | － | － |
| *Neostenanthera myristicifolia* (Oliv.) Exell | Gabon | *Wieringa 3566* (WAG) | AY743486 | AY743467 | EF179306 | － | － | － |
| *Neo-uvaria telopea* Chaowasku | Thailand | *Chaowasku 77* (L) | JX544751 | JX544783 | JX544778 | JX544766 | － | － |
| *Ophrypetalum odoratum* Diels | Kenya | *Robertson 7547* (WAG) | EU169702 | EU169789 | EU169723 | － | － | － |
| *Sanrafaelia ruffonammari* Verdc. | Tanzania | *Kayombo 3027* (MO) | EU169703 | EU169790 | EU169724 | － | － | － |
| *Stenanona costaricensis* R.E. Fr. | Costa Rica | *Chatrou et al. 67* (U) | AY518801 | AY319183 | JX544772 | JX544759 | － | － |
| *Uvaria lucida* Bojer ex Benth. | Cultivated | *Botanische Tuinen 84GR00334* (U) | AY238966 | EF179319 | EF179310 | － | － | － |
| *Xylopia peruviana* R.E. Fr. | Cultivated | *Chatrou 483* (L) | AY238967 | EF179320 | EF179312 | － | － | － |
