# Supplementary material for: Phylogenetic Reconstruction, Morphological Diversification and Generic Delimitation of Disepalum (Annonaceae)
Source: PLoS One. 2015 Dec 2;10(12):e0143481. doi: 10.1371/journal.pone.0143481 (PMC4668016; doi:10.1371/journal.pone.0143481)
Supplement: S2 File — (DOCX) [file pone.0143481.s002.docx]

**S2 File. List of novel primers used for amplification and sequencing of the *AP3*, *ndhF*, and *ycf1* regions.**

| **DNA region** | **Primer name** | **Sequence (5'-3')** |
| --- | --- | --- |
| *AP3* | AP3-1F | AGTTCRGTCAAAGCCGTGCGTG |
|  | AP3-1R | TGCTGTCTGTGGAGATTGAAAACTCT |
|  | AP3-2F | GGCTCAAATCCAATCCCATAACTTGC |
|  | AP3-2R | CCTGCCAACAGTATCATGTCATCAGCA |
| *ndhF* | 1444F | ACAGTGGCACTAAAAACAGTTCCT |
|  | 2060R | AGTTGGGCTAATCCTCTTATACCCC |
| *ycf1* | Ycf1-3450F | TGCGGACACTAGGAATTGCAAAA |
|  | Ycf1-3950R | ACGAGTACAGAGGGTTTGGATAGA |
|  | Ycf1-3880F | TGAGGACAAGAAGGACTCATACTCA |
|  | Ycf1-4390R | ATAAGCATGAGCCGCGGATC |
|  | Ycf1-4280F | ACGGAACCTCGGYYCTTTTGGT |
|  | Ycf1-4582R | GGAAAAAGAACTGTTTAATCAAATGAAGCM |
|  | Ycf1-4358F | ACGGAACCTCGGTTCTTTTGGT |
|  | Ycf1-4986R | ACTCAAGAGGAAAATTCGTCGAGCA |
|  | Ycf1-4502F | TCATAGAAGAAAAAGAAGAGTTTCGTCGT |
|  | Ycf1-5170R | GAGCTACGGAAGTGGAAGAAGTCT |
|  | Ycf1-4897F | AGATGGCGAAAAGGCCAAGGGA |
|  | Ycf1-5468R | TCCAGCATGGGTCGACGAGT |
