# Supplementary material for: Phylogenetic Reconstruction, Morphological Diversification and Generic Delimitation of Disepalum (Annonaceae)
Source: PLoS One. 2015 Dec 2;10(12):e0143481. doi: 10.1371/journal.pone.0143481 (PMC4668016; doi:10.1371/journal.pone.0143481)
Supplement: S3 File — (DOCX) [file pone.0143481.s003.docx]

**S3 File. List of protocols used for amplification of the four cpDNA (*matK*, *trnL-F*, *ndhF* and *ycf1*) and two nDNA (*AP3* and *phyA*) regions.**

|  | **Initial denaturation** |  |  | **Primer** | | |  | **Final extension** |
| --- | --- | --- | --- | --- | --- | --- | --- | --- |
| **Region** |  |  |  | **Denaturation** | **Annealing** | **Extension** |  |  |
|  |  |  |  |  |  |  |  |  |
| *matK* | 94°C for 3 min | 35 cycles of |  | 94°C for 45 s | 52–54°C for 30 s | 72°C for 30 s |  | 74°C for 7 min |
| *trnL-F* | 94°C for 3 min | 35 cycles of |  | 94°C for 45 s | 49°C for 30 s | 72°C for 30 s |  | 74°C for 7 min |
| *ndhF* | 80°C for 5 min | 32 cycles of |  | 95°C for 1 min | 50°C for 1 min, a ramp of 1.3°C s^-1^ until reaching 65°C | 65°C for 4 min |  | 65°C for 5 min |
| *ycf1* | 80°C for 5 min | 32 cycles of |  | 95°C for 1 min | 50°C for 1 min, a ramp of 1.3°C s^-1^ until reaching 65°C | 65°C for 4 min |  | 65°C for 5 min |
| *AP3* | 94°C for 2 min | 38 cycles of |  | 94°C for 30 s | 43°C for 30 s | 72°C for 1 min |  | 74°C for 2 min |
| *phyA* | 95°C for 5 min | 38 cycles of |  | 95°C for 1 min | 60°C for 1 min | 72°C for 1 min 30 s |  | 72°C for 10 min |
|  |  |  |  |  |  |  |  |  |
