# Supplementary figures and images for: Phylogenetic Reconstruction, Morphological Diversification and Generic Delimitation of Disepalum (Annonaceae)
Source: PLoS One. 2015 Dec 2;10(12):e0143481. doi: 10.1371/journal.pone.0143481 (PMC4668016; doi:10.1371/journal.pone.0143481)

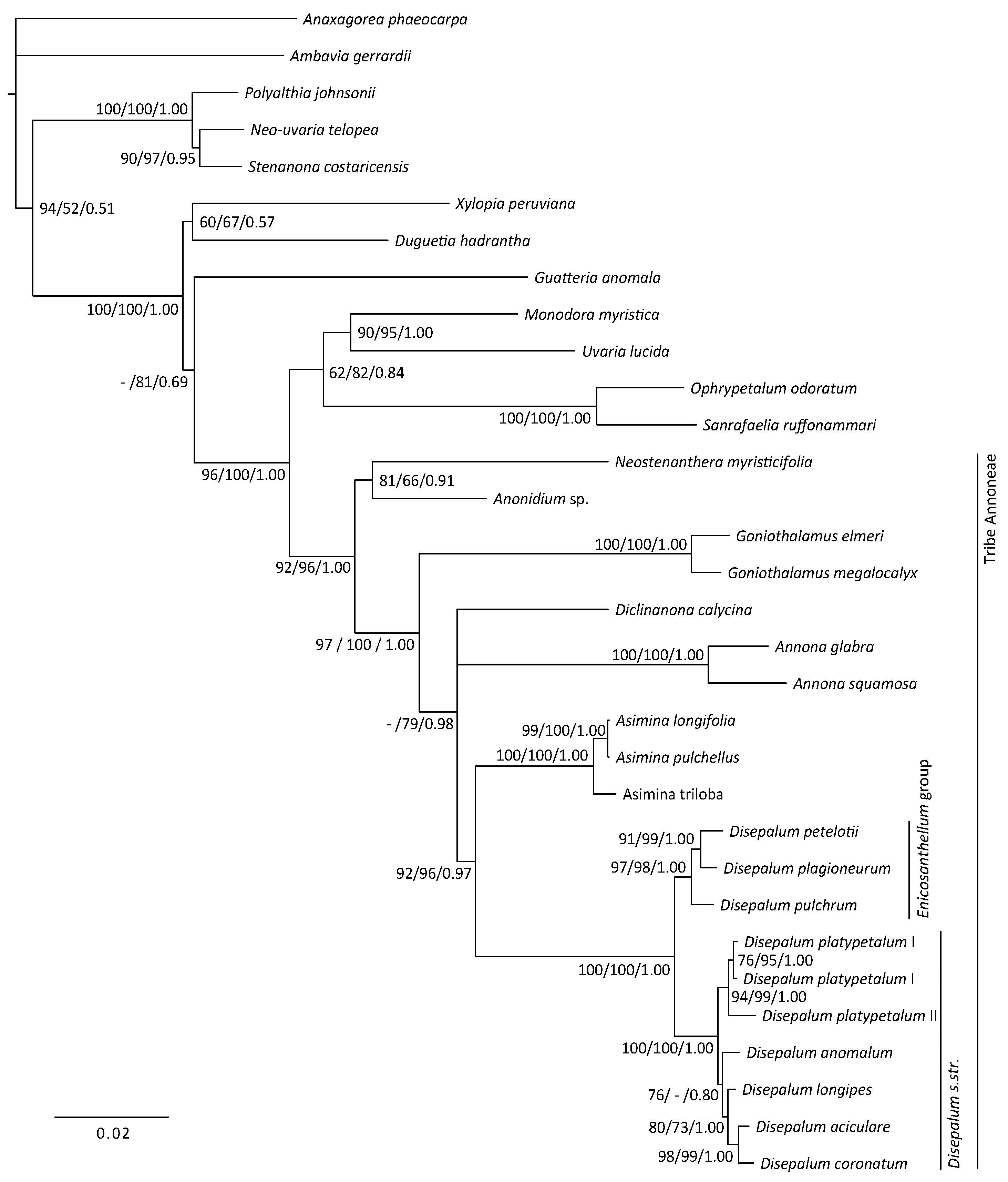

Supplement: S4 File — Numbers at nodes indicate MP, ML bootstrap values (> 50%) and Bayesian posterior probabilities (> 95%). Bootstrap values < 50% are indicated by ‘-’. Scale bar: 0.02 substitutions per site. (TIF) [file pone.0143481.s004.tif]

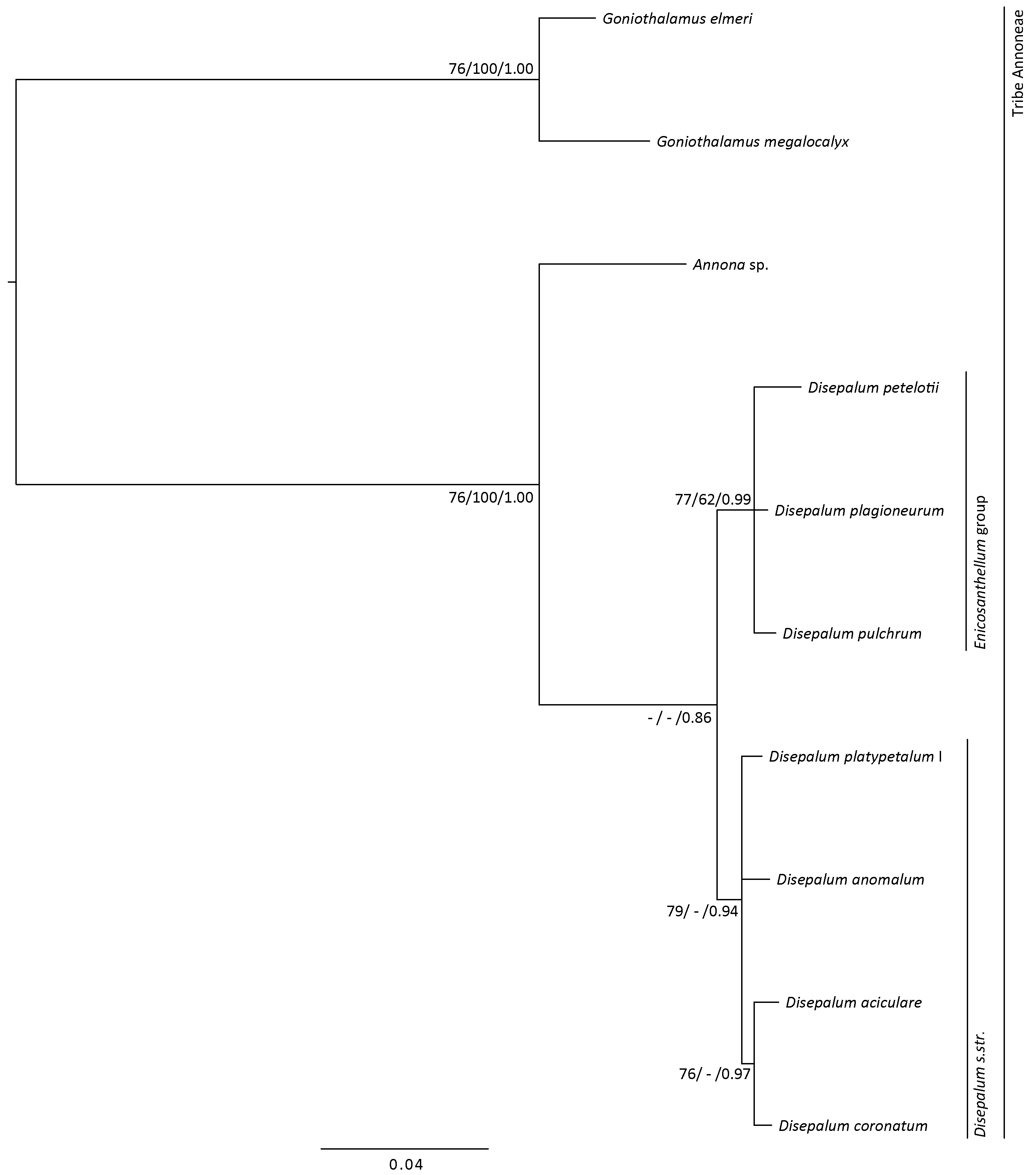

Supplement: S5 File — Numbers at nodes indicate MP, ML bootstrap values (> 50%) and Bayesian posterior probabilities (> 95%). Bootstrap values < 50% are indicated by ‘-’. Scale bar: 0.02 substitutions per site. (TIF) [file pone.0143481.s005.tif]
